# Diaphragmatic dysfunction in patients with acute ischemic stroke and mechanical ventilation

**DOI:** 10.1186/s13054-020-02843-4

**Published:** 2020-04-02

**Authors:** José Vicente Catalá-Ripoll, José Ángel Monsalve-Naharro, Pablo Cuesta-Montero, Francisco Hernández-Fernández

**Affiliations:** 1 Department of Anesthesiology and Critical Care Medicine, Hospital General de Albacete, Complejo Hospitalario Universitario de Albacete, Calle Hermanos Falco, 37, 02006 Albacete, Spain; 2 Department of Neurology, Complejo Hospitalario Universitario de Albacete, Albacete, Spain

Dear Editor,

Neurocritical patient care in intensive care units (ICU) can be challenging. Robba et al. [1] have recently presented a great review where the pathophysiology of brain-lung interactions and the management of mechanical ventilation in these patients with acute ischaemic stroke were explored. A successful extubation prediction of brain-injured patients is complex and challenging, and several factors may lead to weaning failure, including diaphragm dysfunction.

We showed that 51.7% of patients with ischemic stroke can present together diaphragmatic dysfunction contralateral to brain injury [2]. However, it may occur when the cortico-diaphragmatic tract is affected. Diaphragmatic dysfunction usually observed in patients with hemiparesis, although it can also occur in 24% of patients without it. Besides, unilateral diaphragmatic dysfunction can limit contralateral mobility by the bilateral innervation of the diaphragm, causing dyspnea and inspiratory muscle capacity reduction [2]. In addition, mechanical ventilation can lead to a reduced function in both diaphragmatic and intercostal musculature, over the first week of invasive ventilation. An early reduction in diaphragmatic thickness after mechanical ventilation has been observed in up to 50% of patients. This muscular atrophy, which appears mainly in the first week of mechanical ventilation, is associated with prolonged ventilation and increases the risk of unsuccessful extubation [3].

In this context, transthoracic lung ultrasound is increasingly used in the ICU for the bedside assessment of diaphragmatic dysfunction through the thickening fraction [2–5]. This has been proven to be a good dynamic physiological estimator of diaphragm function during mechanical ventilation in a partially assisted system or under pressure support [4]. In addition, the reduction of the thickening fraction with mechanical ventilation has similar strong performance in the prediction of failure of the spontaneous breathing trial [5]. Another validated technique to evaluate the function of the diaphragm is the diaphragmatic excursion, particularly in patients who do not require mechanical ventilation. However, it has greater limitations, for example, in the visualization of the left diaphragm and in the influence of respiratory accessory muscles in the evaluation of the diaphragm. In patients with mechanical ventilation, several studies have been carried out mainly with the thickening fraction [3, 5].

To measure the thickening fraction, a high-frequency probe (7–18 MHz) is placed in the anterior axillary line, trying to obtain the image of the diaphragm between two ribs usually between the seventh and the eighth or the eighth and the ninth. Once the diaphragm is located, a cut is made in M mode; the maximum diaphragmatic thickness is obtained during inspiration, and the minimum during expiration and the thickening fraction is calculated using the formula: [(inspiratory thickness − expiratory thickness)/expiratory thickness]. A value below 0.2 is considered a diagnosis of diaphragmatic dysfunction (Fig. 1) [2], and 0.26 was identified as the optimal threshold value to predict extubation failure [5].

**Fig. 1:**
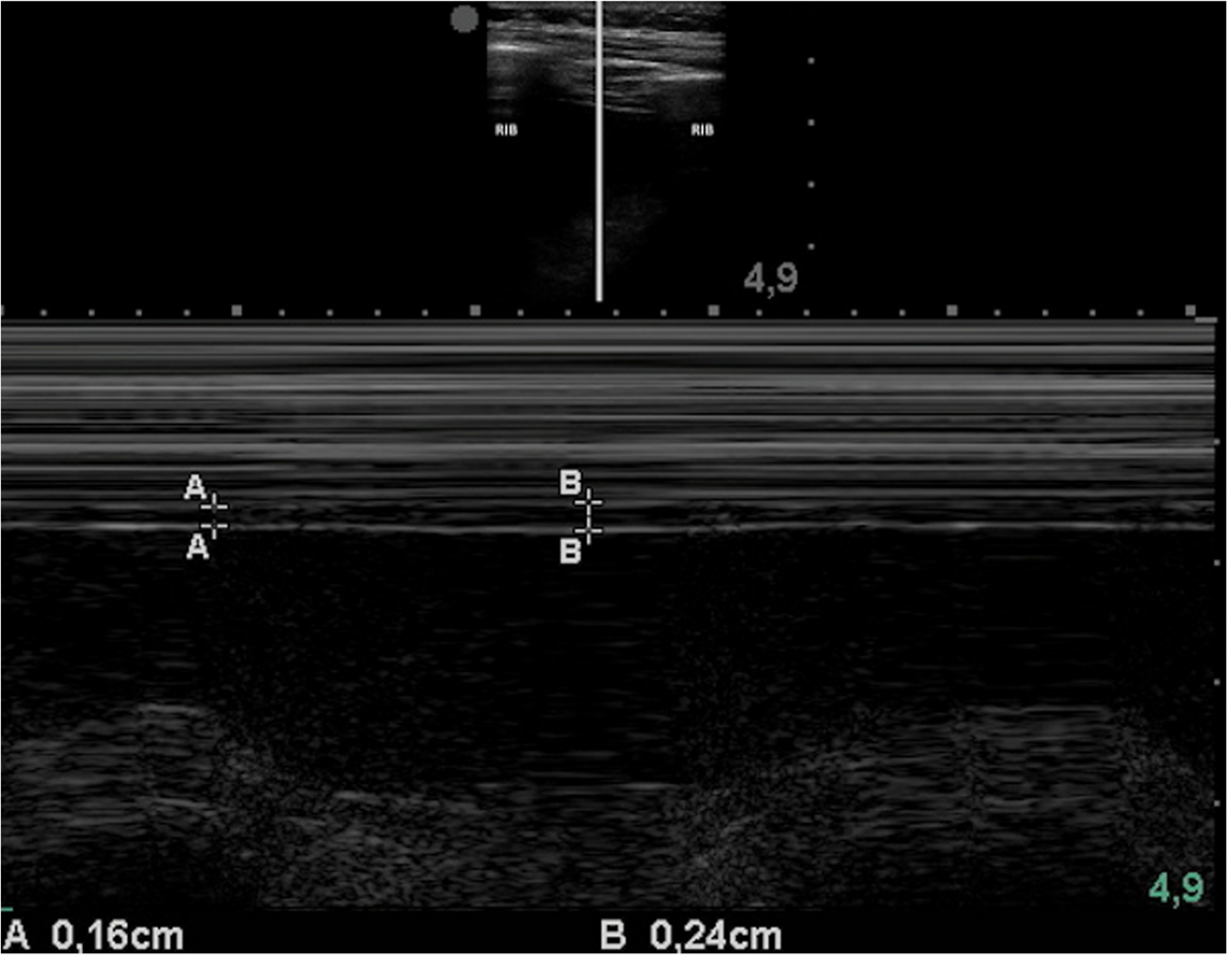
Diaphragmatic M mode. This image shows an increase in diaphragmatic thickness during inspiration. The lines marked with “A” show thickness during expiration, and the lines marked with “B,” the maximum thickness during inspiration

For this reason, diaphragmatic ultrasound is a simple method that allows bedside assessment of diaphragmatic dysfunction as another factor to consider when predicting extubation failure. Further studies to predict the participation of the diaphragmatic dysfunction in the successful weaning of patients with stroke and mechanical ventilation are needed.

## Data Availability

The datasets used and/or analyzed during the current study are available from the corresponding author on reasonable request.
